# The correlation between cancer stem cells and epithelial-mesenchymal transition: molecular mechanisms and significance in cancer theragnosis

**DOI:** 10.3389/fimmu.2024.1417201

**Published:** 2024-09-30

**Authors:** Zi-Ning Lei, Qiu-Xu Teng, Jagadish Koya, Yangruiyu Liu, Zizhou Chen, Leli Zeng, Zhe-Sheng Chen, Shuo Fang, Jinxiang Wang, Yuchen Liu, Yihang Pan

**Affiliations:** ^1^ Scientific Research Center, The Seventh Affiliated Hospital, Sun Yat-sen University, Shenzhen, Guangdong, China; ^2^ Department of Pharmaceutical Sciences, College of Pharmacy and Health Sciences, St. John’s University, New York, NY, United States; ^3^ Big Data Center, The Seventh Affiliated Hospital, Sun Yat-sen University, Shenzhen, Guangdong, China; ^4^ Department of Oncology, The Seventh Affiliated Hospital, Sun Yat-sen University, Shenzhen, Guangdong, China

**Keywords:** cancer stem cells, epithelial-mesenchymal transition, biomarkers, targeted therapy, molecular mechanism

## Abstract

The connections between cancer stem cells (CSCs) and epithelial-mesenchymal transition (EMT) is critical in cancer initiation, progression, metastasis, and therapy resistance, making it a focal point in cancer theragnosis. This review provides a panorama of associations and regulation pathways between CSCs and EMT, highlighting their significance in cancer. The molecular mechanisms underlined EMT are thoroughly explored, including the involvement of key transcription factors and signaling pathways. In addition, the roles of CSCs and EMT in tumor biology and therapy resistance, is further examined in this review. The clinical implications of CSCs-EMT interplay are explored, including identifying mesenchymal-state CSC subpopulations using advanced research methods and developing targeted therapies such as inhibitors and combination treatments. Overall, understanding the reciprocal relationship between EMT and CSCs holds excellent potential for informing the development of personalized therapies and ultimately improving patient outcomes.

## 1 Introduction

Cancer stem cells (CSCs) are a subpopulation with stem cell-like capabilities including self-renewal and differentiation capabilities. These characteristics allow CSCs to be tumorigenic and to shape tumor plasticity and heterogeneity (1). Therefore, CSCs are believed to be involved in tumor initiation, progression, recurrence, metastasis, and therapeutic resistance (2–4). Understanding the unique properties of CSCs has become a focus of cancer research, intending to develop targeted therapies that can effectively eliminate these cells. The CSC theory integrates genomic medicine with the broader context of the tumor microenvironment, emphasizing the interplay between genetic mutations and epigenetic modifications, cellular interactions, and extracellular signals. This holistic approach underscores the importance of considering the tumor as a complex ecosystem, where CSCs interact with other tumor cells and the surrounding stroma (5). Such interactions can influence the behavior of CSCs, including their ability to undergo epithelial-mesenchymal transition (EMT), which is a biological process that allows epithelial cells to acquire mesenchymal, stem cell-like properties, enhancing their migratory capacity, invasiveness, and resistance to apoptosis (6).

**Figure 1:**
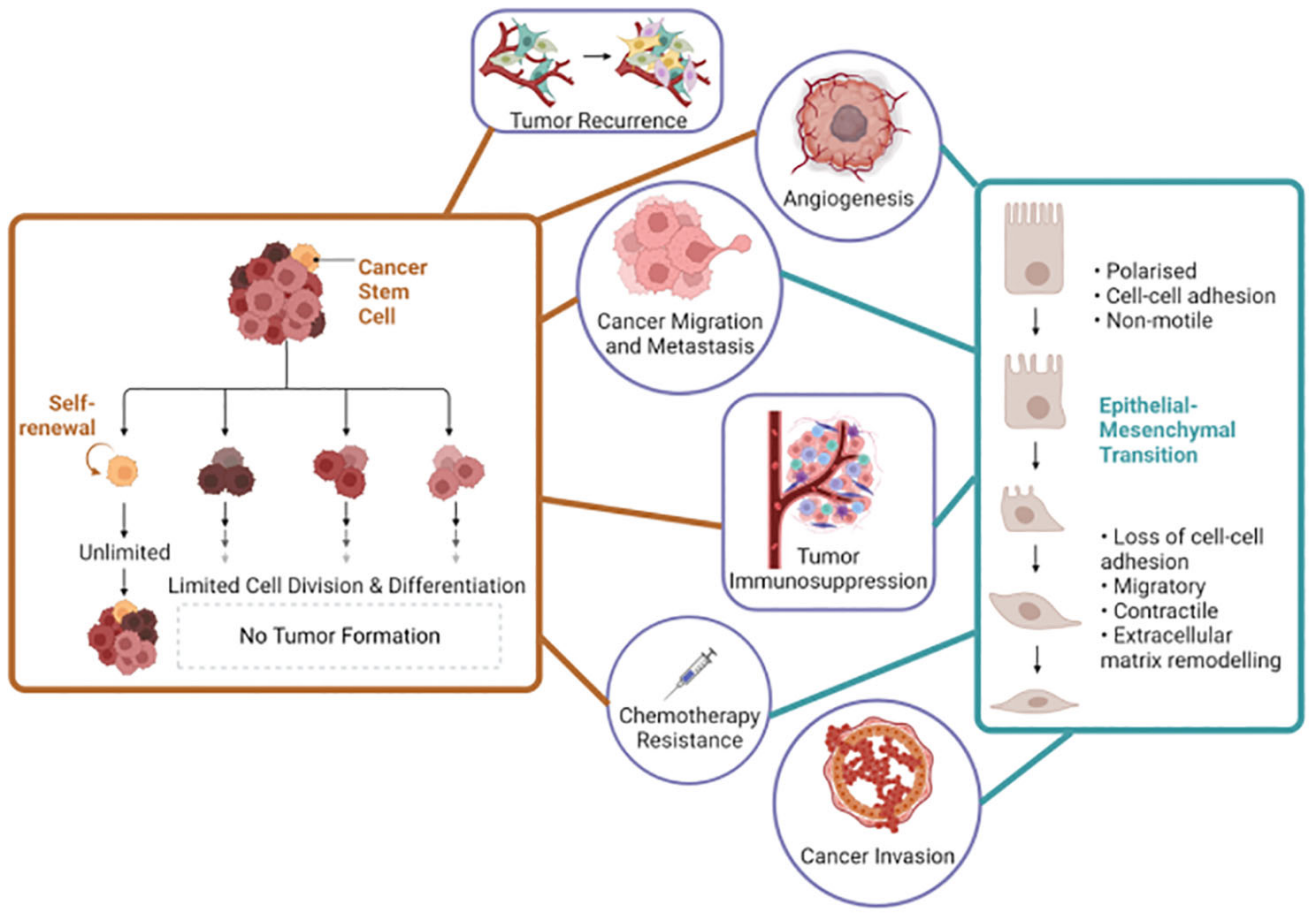
Interconnected roles of cancer stem cells and epithelial-mesenchymal transition in cancer biology. Left side the orange lines represent the key roles of cancer stem cells and the right side the blue lines represent the key roles of epithelial-mesenchymal transition in cancer progression and characteristics. This figure was created with Biorender.com.

The correlation between CSCs and EMT is complex and multifaceted and has garnered substantial attention due to its implication in the orchestration of tumor heterogeneity and malignancy (**Figure 1**). For instance, in triple-negative breast cancer, tumor-associated macrophages have been found to promote EMT and enhance CSC properties via the activation of CCL2/AKT/β-catenin signaling (7). Similarly, Connexin46 expression was found to enhance CSC and EMT properties in human breast cancer MCF-7 cells (8). Furthermore, EMT signaling and CSCs present emerging biomarkers and opportunities for precision therapeutics, particularly in the context of prostate cancer (9, 10). It has become imperative to investigate the intricate relationship between EMT and CSCs in the context of cancer, as this understanding has emerged as a critical area of cancer research with significant implications for developing precision medicine. It is now recognized that EMT contributes to the acquisition of CSC traits in non-CSCs, promoting their self-renewal and differentiation abilities, and enhancing their resistance to therapy (11). Moreover, CSCs have been identified as critical drivers of metastasis, and EMT plays a pivotal role in disseminating these cells from the primary tumor to distant sites (12).

Comprehending the dynamic interplay between EMT and CSCs is crucial for addressing the challenges of tumor heterogeneity, metastasis, and therapy resistance in clinical practice. This understanding forms the foundation for developing innovative, personalized therapeutic approaches that can improve patient outcomes and lead to more effective cancer management. EMT and CSCs are intricately linked, with EMT enhancing CSC properties and contributing to tumor heterogeneity and therapeutic resistance. Key signaling pathways, including TGF-β and Wnt/β-catenin, and transcription factors like Snail and SOX2, drive this interplay, complicating cancer treatment. Personalized medicine, leveraging insights into EMT-CSC dynamics, offers targeted strategies to overcome resistance and improve patient outcomes.

## 2 The interplay between EMT and CSCs

### 2.1 Characteristics of CSCs and EMT

CSCs are studied extensively for their role in tumor initiation, progression, and metastasis. Cancer-specific stem cell growth is regulated with the help of specific microenvironments, and interaction with these microenvironments plays a key role in the maintenance of CSC populations (13). This environment consists of a variety of factors such as hypoxic regions, stromal cells, immune cells, network of cytokines and growth factors, and the extracellular matrix (ECM), which helps in supporting the stemness of these CSCs by modifying the self-renewal pathways such as Wnt/*β*-catenin, Notch, NF-κB, JAK/STAT and Hedgehog or by hindering the transcriptional regulators such as NANOG, OCT-4, and SOX-2 (13–16). CSC-enriched populations can be distinguished by identifying specific normal stem cell markers and some distinct markers such as CD133, CD24, CD44, EpCAM (epithelial cell adhesion molecule), CD117, THY1, ALDH1, and CD200 when they are isolated from solid or hematological tumor tissues (16–21). Certain cancer cells have the flexibility to undergo a reversible dedifferentiation process, such as epithelial to mesenchymal transition(EMT), and convert from a differentiated state to a stem cell-like state depending on the distinct environmental stimulus (11). These cancer cells that have undergone EMT become more invasive and metastatic due to the presence of self-renewal EMT-mediating transcription factors such as Snail, Zebi, SOX2, KLF4, and many others which were discussed extensively before (13, 22). Numerous cellular processes, such as migration, metastasis, invasion, ECM alteration, and apoptosis, were controlled by EMT (12). EMT markers and stem cell markers (**Table 1**) are found to be expressed in circulating tumor cells in metastasized patients, which makes them potential targets for treating cancer in a targeted approach (52).

**Table 1:** EMT and CSCs representative biomarkers and the principle signaling pathway involved.

Cancer Type	Biomarkers for EMT	Biomarkers for CSC	Principle Signaling Pathway
Lung Cancer	Snail (23), Slug, Twist (24)	CD44+CD24- (25), CD133+ (26)	TGF-β/Smad, Wnt/β-catenin
Liver Cancer	β-catenin (27)	CD133+ (28), CD13+ (29), CD45-CD90+ (18)	Wnt/β-catenin, Notch
Esophageal Cancer	Nanog, SOX2 (30)	ALDH1A1+ (31)	Wnt/β-catenin, PTEN/PI3K/AKT
Breast Cancer	Notch1, Jagged 1 (32)	ALDH1A1+ (33), CD44+CD24- (34)	Notch, PTEN
Gastric Cancer	GSK-3β, Snail, Slug, Twist (35)	CD44+, CD133+, ALDH1A1+ (36)	Wnt/β-catenin, Hedgehog
Pancreas Cancer	Snail, Twist, SOX4 (37)	CD133+ (38), CD105+ (39)	Wnt/β-catenin, Hedgehog
Colon Cancer	E-cadherin, β-catenin (40)	Lgr5+ (41)	Wnt/β-catenin
Renal Cancer	Slug, LEF (42)	CD133+ (43), CD105+ (44)	Wnt/β-catenin, Hedgehog
Prostate Cancer	β-catenin (45), CXCR4 (46)	CD44+ (47)	TGF-β/Smad, Hedgehog Wnt/β-catenin,
Brain Cancer	MMP1, VIM (48)	CD133+, CD44+ (49)	TGF-β/ Smad, Notch
Leukemia	AKT, VIM (50)	CD133+, CD34+/CD38- (51)	TGF-β/Smad, Hedgehog Wnt/β-catenin,

EMT is an intricate and highly dynamic cellular process that assumes a pivotal role in various physiological and pathological contexts (53), encompassing embryonic development (54), chronic inflammation (55), fibrotic diseases like renal fibrosis (56), as well as cancer progression and metastasis (57). Traditionally, epithelial cells were characterized as terminally differentiated with distinct apical-basal polarity (58)serving protective, supportive, and secretory roles (59, 60). However, recent findings show that epithelial cells can undergo a series of changes that entail the loss of cell polarity (61), disintegration of intercellular tight junctions and adherent junctions, acquisition of migratory capabilities (62, 63), and the adoption of mesenchymal cell morphology and attributes (64), culminating in what is defined as EMT. Therefore, EMT is mainly characterized by the loss of polarity of epithelial cells and the acquisition of mesenchymal properties (65), including fibroblast-like appearance (66) and the upregulation of genes like vimentin (67, 68), Snail (69), and osteopontin (70). Consequently, cells transition towards a fibroblast-like morphology or outright transformation into mesenchymal cells, accompanied by enhanced migration and metastatic potential (71). It is a complex dynamic process, mainly manifested as, on the one hand, it occurs between cells, leading to the relaxation of tight cell-cell junctions and ECM degradation through enzymatic hydrolysis (63); on the other hand, it occurs inside cells, involving profound cytoskeletal reorganization, diminished keratin expression, actin filament restructuring, and the induction of vimentin expression, ultimately morphing cells into spindle-shaped fibroblast-like entities (66, 68). These changes collectively amplify cellular migratory and invasive capacities, thus constituting a foundation for physiological and pathological phenomena.

### 2.2 Role of CSCs and EMT in tumor initiation, progression and metastasis

Previous literature has speculated that even one CSC can be enough to regenerate a tumor (6). However, in order to maintain their stemness, CSCs required to be in exposure of specialized microenvironments also known as stem cell niches (13, 72). Several niches, such as hypoxic conditions, vascular niche etc, were studied extensively to understand how these microenvironments were aiding in maintaining the CSCs and reprogramming normal cancer cells to CSCs thereby promoting more tumor formations. One such example of vascular niche involvement was a study that has demonstrated CSC populations expressing CD133+ marker in brain, liver, and pancreatic cancers produce increased levels of vascular endothelial growth factor (VEGF) and stromal-derived factor-1 (SDF-1), which stimulate angiogenesis thereby promoting metastasis (73–75). Accumulating evidence shows EMT is associated with many signaling pathways such as androgen receptor signaling, estrogen receptor signaling, TGF-β (transforming growth factor β) signaling, epidermal growth factor (EGF) signaling, Sonic hedgehog and WNT signaling pathways and its role in the related tissue development, wound healing and cancer (76, 77). Self-renewal pathways such as NF-κB and the Wnt/β-catenin pathway were shown to contribute to EMT activation, which is directly related to increased cancer invasiveness and aggressiveness, resulting in poor patient outcomes (20, 78). The interplay between CSC and EMT-involved pathways was regulated at a genetic level (79).

In particular scenarios like breast cancer, CSCs identified as BCSCs (Breast cancer stem cells) exist in specific development states, the first one being in a mesenchymal state expressing CD24-/CD44+ markers on their cell surface and the second being in an epithelial cell-like state expressing an enzyme known as Aldehyde dehydrogenase (ALDH) (80). A more significant tumor-initiating capacity was observed in BCSCs expressing both the marker and enzyme profiles (81).

### 2.3 Alteration of tumor heterogeneity, plasticity and tumor microenvironment alteration

Numerous literatures have shown that tumors contain explicit cell populations that vary in multiple factors like karyotype, phenotype, and chemoresistance (82–84). Sequencing studies have shown distinct epigenetic characteristics of multiple cell clones within the same tumor (85). This phenomenon is termed tumor heterogeneity and is primarily orchestrated by producing various phenotypically different subclones inside the tumor mass by CSCs (86–88). EMT also contributes to cell plasticity and promotes intra-tumor heterogeneity (89, 90).

Both EMT and CSC properties were related in tumors exhibiting resistance to cytotoxic T-lymphocytes (91). Tumor-associated macrophages (TAMs), a part of the stem niche in the tumor microenvironment, contributed to EMT characterization (92). Other studies have also indicated that TAMs can induce chemoresistance in myeloma cell lines by directly interacting with malignant cells and inhibiting the activation of caspase-dependent apoptotic signaling (19).

Overexpression of drug transporter proteins of the ABC family (mainly MDR1, ABCG2, and ABCB1) on the cell surface of various CSCs has been shown to contribute to chemo-drug resistance and promote disease relapse (93, 94). Self-renewal and cell proliferation signaling pathways like JNK were shown to be upregulated in CSCs and contribute towards cancer resistance by reducing the generation of intracellular ROS caused by 5-fluorouracil and gemcitabine (95, 96).

## 3 Molecular mechanisms and regulatory factors of EMT

### 3.1 Characteristics of EMT

The regulation of EMT involves a variety of molecular mechanisms and regulatory factors. Several key signaling pathways and transcription factors have been identified as central players in controlling EMT and its association with cancer stem cells (CSCs). For example, transforming growth factor-β (TGF-β) signaling is a potent inducer of EMT, promoting downregulation of E-cadherin and upregulation of N-cadherin and vimentin, thereby driving cells toward a mesenchymal phenotype development (97, 98). In addition, the Wnt/β-catenin pathway activates EMT-associated transcription factors such as Snail and Twist, suppressing epithelial markers and acquiring stem cell-like properties (99, 100). Furthermore, transcription factors such as Nanog, OCT4, SOX2, and KLF4, which are critical for maintaining stem cell pluripotency, have been found to interact with EMT regulators to enhance the stem-like characteristics of cancer cells (101–103).

Tumors are composed of heterogeneous cell populations. Under normal circumstances, maintaining the homeostasis of various tissues and organs in the body is based on self-renewal, multidirectional differentiation and proliferation of CSCs are all modulated by intricate regulatory mechanisms. However, the interplay of various stimulating factors *in vivo* and *in vitro*, in conjunction with the influence of CSCs, can trigger genetic mutations, promoting the development of tumors (5). In fact, CSCs tend to accumulate numerous gene mutations, which lead to the loss of normal regulation of cells, excessive proliferation, and even metastasis (2–4). Essential signaling pathways governing cellular division and differentiation, such as Notch, Wnt/β-Catenin, Ras/Raf/Mek/Erk, exert substantial influence within the context of CSCs (104). Altered or hyperactive signaling through any of these pathways can disrupt standard cellular regulatory mechanisms, leading to the occurrence of tumors (105). The complex network of molecular mechanisms and regulatory factors that control EMT and its association with CSCs highlights the complexity of this process and its importance in cancer biology.

### 3.2 Signaling pathways and transcription factors regulating EMT and CSCs

**Figure 2:**
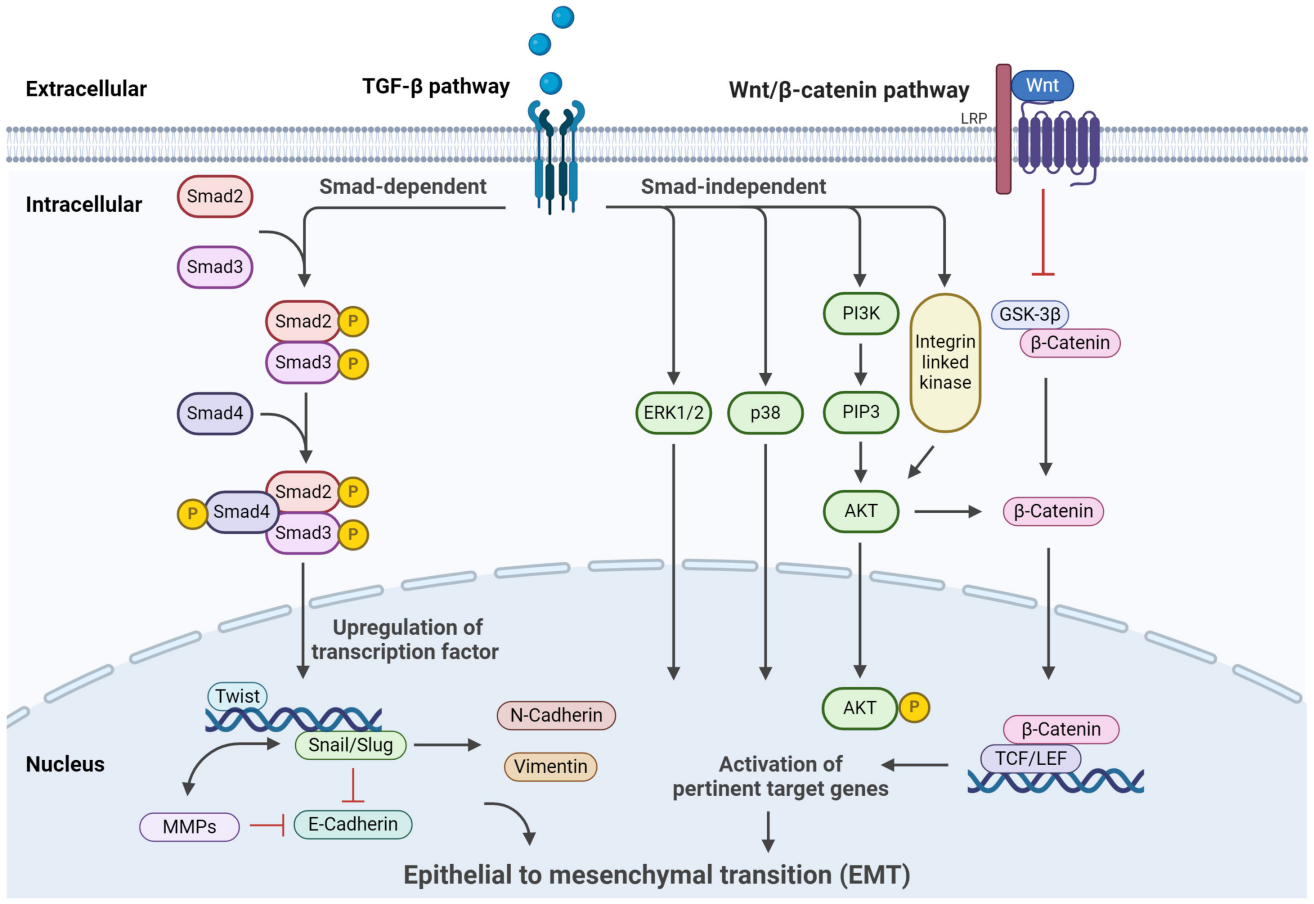
Signaling pathways regulating EMT. The primary signaling and crosstalk of the TGF-β/Smad signaling pathway, Wnt/β-catenin signaling pathway, Hedgehog signaling pathways, and their regulatory roles in cellular processes are illustrated. This figure was created with Biorender.com.

EMT is a complex process regulated by a network of signaling pathways and transcription factors intricately involved in controlling CSCs (**Figure 2**). Among these pathways, TGF-β stands out as a prominent inducer of EMT in various cancer types. TGF-β activates downstream Smad signaling, leading to upregulating transcription factors such as Snail, Slug, and Twist (106). These EMT-associated transcription factors actively downregulate E-cadherin while promoting the expression of mesenchymal markers, including N-cadherin and vimentin (107–109). Consequently, the epithelial cells undergo a metamorphosis toward a mesenchymal phenotype. TGF-β is paramount in EMT, often serving as a positive control in inducing EMT in experimental setups (109). In addition, TGF-β can enhance the self-renewal ability of CSCs, contributing to tumor progression and treatment resistance. As a pleiotropic cytokine, TGF-β exists in three subtypes: TGF-β1, TGF-β2, and TGF-β3 (110), with TGF-β1 being the extensively studied variant. Its presence is widespread in diverse tumors, exerting regulatory effects on diverse cellular processes such as immunosuppression, growth inhibition, EMT, and cell migration (111, 112). The role of TGF-β varies throughout tumorigenesis; in the early stages, it operates as a tumor suppressor, while in advanced tumors, it facilitates tumor growth (113, 114). TGF-β-induced EMT signaling pathways in tumor cells can be divided into two types according to whether Smad protein is involved, Smad-dependent classic TGF-β signaling pathway and Smad-independent TGF-β signaling pathway. In the TGF-β/Smad signaling pathway, TGF-β binds to specific receptors on the cell membrane, inducing phosphorylation of Smad2/Smad3, followed by heterotrimer formation with Smad4 (115). This complex translocate to the nucleus, upregulating transcription factors like Snail/Slug and Twist (106). Concurrently, E-cadherin expression is downregulated, effectively controlling EMT at the transcriptional level (97). For Smad-independent signaling pathways, on the other hand, alternate EMT-associated pathways like integrin, PI3K/AKT, and mitogen-activated protein kinase (MAPK) (116). TGF-β’s interaction with its transmembrane receptor activates PI3K, which generates the secondary messenger PIP3, subsequently phosphorylating AKT. This leads to AKT translocating to the nucleus, hindering GSK-3β-mediated degradation of β-catenin or activating nuclear factor-κB, thereby influencing EMT (117, 118). In addition to the PI3K/AKT signaling pathway, TGF-β’s involvement extends to the Ras-Raf-MAPK pathway, where downstream transcription factors, including extracellular signal-regulated kinases 1/2 (ERK1/2) and p38MAPK, facilitate EMT by modulating downstream target gene transcription (116).

The Wnt/β-catenin pathway emerges as a pivotal regulator of EMT and CSC. In the Wnt/β-catenin signaling pathway, the Wnt protein binds to the corresponding transmembrane receptors, thereby inhibiting GSK-3β activity (118). This stymies the degradation of β-catenin, resulting in its intracellular accumulation. When β-catenin accumulates to a certain extent, it enters the nucleus, which collaborates with lymphoid enhancer factor/T cell factor (LEF/TCF) transcription factors to activate genes pertinent to EMT (119, 120). This sequence leads to the suppression of E-cadherin expression and facilitates EMT progression. In the process of EMT, an important molecular event is the downregulation of E-cadherin, and the transcription factor Snail can bind to the E-cadherin promoter region to inhibit the expression of E-cadherin (121). E-cadherin is a typical single transmembrane glycoprotein, which can regulate the adhesion between Ca^2+^-dependent cells and play an essential role in cell polarization and tissue formation (122). Downregulation or inhibition of E-cadherin expression will turn on EMT and lead to tumor invasion and metastasis. Batlle et al. determined that in many tumors, E-cadherin is considered to be the target gene directly acted by Snail, which directly binds to the E-box sequence of the promoter region of E-cadherin, which reduces the expression or defect of E-cadherin, thereby triggering EMT (123). In addition, Zhang et al. found that antisense Snail prevents the occurrence of EMT, and the process can be reversed by silencing the expression of Snail protein (124). Furthermore, the transcription factor Snail can indirectly upregulate the expression of matrix metalloproteinase (MMPS) family members MMP-1, MMP-2, and MMP-7, inhibit matrix synthesis, accelerate matrix component decomposition, and increase cell invasion ability (125). Therefore, Snail induces EMT and plays an essential role in tumor invasion and metastasis. Indeed, the significance of Snail’s involvement extends beyond EMT, encompassing various processes within tumor initiation and embryonic morphogenesis (126). Snail can initiate EMT from different pathways, and Snail plays a central role in this process, coordinating the induction of different signaling pathways. Experiments have observed that normal stem cell proliferation is disordered when the Wnt pathway is abnormally activated, and even tumor proliferation is formed. On the contrary, Fatima et al. shut down the Wnt signaling pathway of cells, reduced the content of β-catenin in liver cancer cells, and observed that the proliferation of liver cancer cells was weakened (127, 128). The Wnt signaling pathway plays a crucial role in colorectal carcinogenesis and has emerged as a target for stem cell therapy against colon cancer.

Moreover, the process of EMT is also affected by transcription factors that play an essential role in stem cell pluripotency. These factors, including Nanog, OCT4, SOX2, and KLF4, constitute pivotal determinants for maintaining the undifferentiated state of embryonic stem cells (101–103). Aberrant expression of these factors within the context of cancer cells can profoundly propel EMT processes and promote CSC properties. Specifically, Nanog, emerging as a standout influencer, has been shown to directly regulate EMT-related genes and promote the stemness of cancer cells (101, 102). The Notch pathway is highly conserved in the process of biological evolution, comprising Notch receptors (Notch1-4), Notch ligands (Jagged 1, Jagged 2, Delta-likeligand1-4) and CSL (DNA binding protein), dividing into CSL-dependent pathway and CSL-independent pathway (129). Its primary roles encompass the maintenance of stem cell existence and initiating postnatal embryonic or fetal cell differentiation. The Notch pathway affects cell differentiation, proliferation, and apoptosis, constituting a pivotal determinant in the genesis of malignant tumors (130). Likewise, OCT4 and SOX2 have been discerned to interact collaboratively with EMT transcription factors, thereby intensifying cancer cells’ plasticity and stem-like attributes. The hedgehog signaling pathway is essential in CSCs, mainly in basal cell tumors (131). This pathway is regulated by two receptors, namely Patched and Smoothened (SMO), positioned on the target cell membrane (132). Patched receptors inhibit the activation of the Hedgehog pathway, while SMO receptors promote the activation of the Hedgehog pathway. Activation of SMO receptors triggers downstream Hedgehog target gene activation, thereby delineating an intricate regulatory landscape governing cellular proliferation and survival (133, 134).

The intricate interplay among signaling pathways, such as TGF-β and Wnt/β-catenin, along with transcription factors including Snail, Nanog, OCT4, SOX2, and KLF4, controls the EMT process and the development of CSCs. Many factors regulate the occurrence of EMT in tumor cells. After undergoing EMT, tumor cells can migrate to adjacent organs or distant sites, concurrently conferring resistance against conventional chemotherapy and drug treatment. Understanding the convoluted regulatory networks that control EMT and CSCs can provide valuable insights into tumor progression, metastasis, and resistance to therapeutic interventions. However, studies have shown that EMT is not an irreversible process, and reversing or inhibiting EMT may be an effective way to inhibit tumor cell migration or distant metastasis (135). Targeting these pathways and factors holds great promise in developing novel and personalized cancer therapies. Further research in this area will help advance our knowledge and improve clinical outcomes for cancer patients.

## 4 Clinical implications of EMT and CSCs: biomarkers and targeted therapies

The research on EMT-related signaling pathways provides a direction for treating tumors and has also become the main target of clinical new drug development. Studies have found that integrin-linked kinase can activate AKT to lead to EMT, and down-regulating integrin-linked kinase can inhibit EMT and metastasis of tumor cells in endocrine cancer (136). Similarly, cysteine protease inhibitor C (CystC) modulates the TGF-β signaling pathway across both normal and tumor cells (137). The Ras pathway also suggests targets for the research of tumor therapeutic drugs. For example, farnesyltransferase inhibitors play an anti-tumor effect by inhibiting the binding of Ras to the cell membrane and have been applied to the treatment of various tumors (138). Src kinase inhibitors such as dasatinib can effectively inhibit the growth of cells undergoing EMT, thereby inhibiting tumor growth (139). Based on the close relationship between EMT and all aspects of tumor metastasis, designing targeted intervention strategies for the core regulatory mechanisms of EMT has become a research hotspot in metastasis treatment. The initial strategy to intervene in EMT is to directly block or reverse EMT to allow cells to regain their epithelial cell characteristics. This is exemplified by interventions like ADH-1 targeting N-cadherin (140), Fresolimumab targeting TGF-β (141), Catumaxomab targeting EpCAM (142), and GN-25 targeting Snail (143).

Furthermore, the landscape of tumor therapy has been redefined through the advent of RNA interference and (miRNA) silencing strategies. For example, the targeted silencing of Snail gene expression through short hairpin RNA has demonstrated the potential to reverse EMT and inhibit *in vivo* tumor growth (144). Similarly, the utilization of small interfering RNA targeting TGF-β has shown promise in attenuating tumor metastasis *in vivo*, substantiating the prospect of impeding EMT initiation via the inhibition of TGF-β secretion within the tumor cell matrix. Krutzfeldt et al. proposed that precise silencing of endogenous miRNAs and RNA antagonists could also silence specific miRNA expressions *in vivo* (145). Therefore, using miRNA as a therapeutic target to inhibit the occurrence of EMT is also a new strategy for tumor treatment.

Further research on EMT-related regulatory factors will help better understand the mechanism of tumor cell EMT and provide a new direction for understanding the mechanism of tumor metastasis and recurrence. Consequently, the multifaceted research imparts novel insights with the potential to reshape the approach to tumor treatment. Reducing the drug resistance of tumor cells by inhibiting EMT of tumor cells can be used as a supplement to conventional tumor therapy and become a new personalized tumor treatment method. Developing new anti-tumor drugs targeting EMT will be a new research direction.

Currently, the conventional cancer treatment modalities encompass surgical excision, chemotherapy, radiotherapy, ablation, and a combination of these methodologies. While these approaches have substantially enhanced the overall survival rate of cancer patients, the persisting challenge remains cancer recurrence. Although traditional approaches manage to eliminate the bulk of cancer cells, the enduring presence of cancer stem cells (CSCs), constituting a minute fraction of the total cellular population, invariably leads to cancer recurrence. The comprehensive eradication of these CSCs is a pivotal requisite for attaining curative outcomes. Consequently, CSCs are recognized as the origin of the occurrence and development of malignant tumors. By dissecting the attributes of CSCs, the potential emerges to elucidate the pertinent CSC surface markers, formulate tailored therapeutics targeting specific entities, and unearth novel avenues for malignancy management. In clinical spheres, the development of therapeutics and treatments singularly aimed at CSCs has gained traction, thereby conferring a degree of selectivity towards CSCs while preserving the homeostasis of normal stem cells. This selection arises from the observation that CSCs, equipped with ATP-binding cassette (ABC) transporters, can efflux drugs from their cytoplasm, thereby fortifying themselves against drug toxicity, endowing them with an inherent resistance to conventional chemotherapeutics and radiation therapy, along with a propensity for angiogenesis promotion.

Compared with paclitaxel, a commonly used chemotherapeutic drug for breast cancer, salinomycin demonstrated a significantly greater inhibitory effect on the growth of murine mammary tumors in breast cancer xenografted nude mice (146, 147). Salinomycin achieved this by targeting the EMT processes crucial for CSC maintenance and metastasis (148). Its efficacy is about 100 times greater than that of paclitaxel, due to its ability to inhibit EMT, thereby reducing the expression of key EMT markers such as Snail, Slug, and Twist (84, 149, 150). This process diminishes the CSC population, which is essential for tumor initiation, progression, and metastasis (151). Notably, the absence of breast cancer stem cell-associated gene expression, particularly the CD44+CD24- phenotype was observed with salinomycin treatment, further emphasizing its specific action against CSCs (152, 153). This heightened effect correlates with downregulating genes critical for CSC function, such as ALDH1 (154) and the ABC transporter genes (155, 156). Additionally, studies have shown that salinomycin disrupts the Wnt/β-catenin signaling pathway, which is often upregulated in CSCs, thereby enhancing its ability to target and eliminate these cells (157, 158). In contrast, paclitaxel primarily targets rapidly dividing cells but does not specifically affect the EMT or CSC pathways, which contributes to its reduced efficacy against CSCs (159, 160). Meanwhile, studies reported the capacity of pancreatic stem cells to undergo differentiation into functional pancreatic cells post-transplantation and play an essential role in the recovery of pancreatic endocrine function and the repair of pancreatic cell damage (161, 162). In addition, a small molecule Hedgehog signaling pathway inhibitor, cyclopamine, reduced acetaldehyde dehydrogenase by preventing aberrant pancreatic cancer Hedgehog signaling, which is often linked to CSC maintenance (163). This pharmacological approach presents promise in pancreatic cancer therapy by targeting pathways essential for CSC survival and EMT, similar to the mechanism of action of salinomycin in breast cancer.

Studies have shown that miRNA, combined with suitable carriers, can repair multiple disease-related abnormal pathways by restoring miRNA expression and targeting CSC (164), offering a novel approach to tumor therapy. *In vitro* assays and xenograft mice models demonstrated the effectiveness of inhibitors targeting signaling pathways like Wnt/β-catenin, Notch, and Hedgehog in effectively inhibiting CSC self-renewal (165, 166). The future focus of CSC research must be the development of precision drugs designed to selectively kill tumor cells to achieve the goal of eradicating tumors.

## 5 Summary

A growing body of research has illuminated the intricate and reciprocal relationship between EMT and CSCs, shedding light on their profound implications for cancer biology. EMT promotes CSC properties in non-CSCs, leading to tumor heterogeneity and enhanced metastatic potential (89, 90). Notably, EMT-induced phenotypic changes, including the loss of epithelial characteristics and the acquisition of mesenchymal traits, parallel the emergence of CSC traits (2–4). Moreover, the emergence of CSCs through EMT is closely linked to therapy resistance, as CSCs are known to be resilient to conventional treatments. EMT-driven CSCs often exhibit increased drug efflux mechanisms and the ability to evade the cytotoxic effects of therapeutic agents, presenting a formidable challenge in clinical oncology (93, 94).

Further investigations have unveiled the regulatory networks that govern the crosstalk between EMT and CSCs, highlighting key signaling pathways and transcription factors that serve as central orchestrators (97, 98). The TGF-β and Wnt/β-catenin pathways, among others, have been identified as critical inducers of EMT and are closely linked to CSC properties. Moreover, transcription factors such as Snail, Slug, Nanog, OCT4, SOX2, and KLF4 play pivotal roles in driving EMT-associated transformations and enhancing CSC properties (101–103). This intricate web of molecular mechanisms underscores the complexity of the EMT-CSC interplay. Disrupting this interplay is crucial for cancer therapy, potentially overcoming therapeutic resistance.

Additionally, the emergence of personalized medicine is intimately linked to understanding the EMT-CSC relationship. Through advanced research methods, it is now feasible to identify EMT-CSC subpopulations within individual tumors (167, 168). This provides a foundation for developing tailored treatments targeting a patient’s tumor’s specific molecular and cellular characteristics. Personalized therapies may involve a combination of EMT and CSC-targeted agents to maximize treatment efficacy while minimizing side effects.

In conclusion, the reciprocal relationship between EMT and CSCs has profound implications for developing targeted cancer therapies and personalized medicine. By disrupting this intricate interplay, we have the potential to transform the landscape of cancer treatment, offering new hope for patients and improving clinical outcomes.
